# Mimetic peptide of ubiquitin-interacting motif of epsin as a cancer therapeutic-perspective in brain tumor therapy through regulating VEGFR2 signaling

**DOI:** 10.20517/2574-1209.2016.01

**Published:** 2017-03-31

**Authors:** Yunzhou Dong, Hao Wu, Jerry Dong, Kai Song, Habibunnabi Ashiqur Rahman, Rheal Towner, Hong Chen

**Affiliations:** 1 Vascular Biology Program, Department of Surgery, Boston Children’s Hospital, Harvard Medical School, Boston, MA 02115, USA; 2 Advanced Magnetic Resonance Center, Oklahoma Medical Research Foundation, Oklahoma, OK 73104, USA

**Keywords:** UPI peptide, epsin, cancer therapy, glioma, tumor angiogenesis

## Abstract

Epsins, endocytic adaptor proteins required for internalization of ubiquitylated receptors, are generally upregulated in human cancers. It has been characterized that mice deficient of epsins in the endothelium inhibit tumor growth by dysregulating vascular endothelial growth factor receptor-2 (VEGFR2) signaling and non-productive tumor angiogenesis. Binding of the epsin ubiquitin (Ub)-interacting motif (UIM) with ubiquitylated VEGFR2 is a critical mechanism for epsin-dependent VEGFR2 endocytosis and degradation, indicative of epsin UIM as a potential therapeutic target. A Computer Assisted Drug Design approach was utilized to create the UIM mimetic peptides for the functional competition of epsin binding sites in ubiquitylated VEGFR2 *in vivo*. Specifically targeting VEGFR2 in the tumor vasculature, the chemically synthesized chimeric UIM peptide, UPI, causes non-functional tumor angiogenesis, retards tumor growth, and increases survival rates in several tumor models. The authors showed that UPI binds ubiquitylated VEGFR2 to form a supercomplex in an Ub-dependent fashion. Collectively, the UPI targeting strategy offers a potentially novel treatment for cancer patients who are resistant to current anti-angiogenic therapies. In this review, the authors outline the main points of this research specifically as a potential application for glioma tumor therapy.

## INTRODUCTION

Angiogenesis is essential for embryogenesis and postnatal tissue repair. Deadly cancers, however, can emerge from tumor angiogenesis, a physiological process involving the production of functional vessels for cancer cell embedment, colonization, growth, and metastasis. In 1971, Folkman^[1]^ hypothesized that inhibition of tumor angiogenesis is a potentially powerful tool for cancer therapy.^[2–6]^ With the identification of more and more new molecules modulating angiogenesis,^[6–8]^ targeting tumor angiogenesis has become increasingly likely, and the concept of inhibiting tumor vessel growth has led to the discovery of vascular endothelial growth factor (VEGF) and the anti-VEGF antibody, Bevacizumab (Avastin). Bevacizumab is an angiogenic inhibitor approved by the U.S. Food and Drug Administration (FDA) for certain metastatic cancers such as colorectal cancer and lung cancers.^[9]^ However, this approach centered around the major pathways including vascular endothelial growth factor receptor (VEGFR) or Notch signaling via direct or indirect modulations.^[5,10–13]^ Because the therapeutic efficacy of Bevacizumab is mild in clinical applications where patients could develop resistance to the drug during the course of the treatment, it was imperative to develop alternative compounds to modulate tumor angiogenesis and complement the efficacy of Bevacizumab for those who are resistant to anti-angiogenic therapies.

## EPSIN UBIQUITIN-INTERACTING MOTIF AS A THERAPEUTIC TARGET FOR CANCER

### Epsins are adaptor proteins in endocytosis

**Figure 1:**
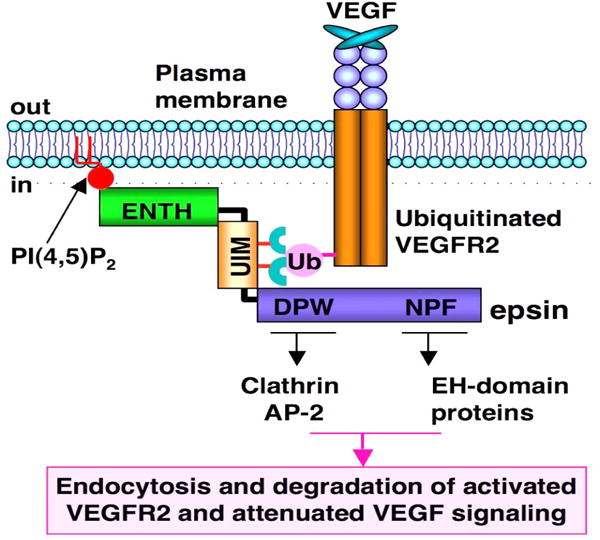
Epsins are adaptor proteins in endocytosis. VEGFR2 is activated by ligand-VEGF binding. Activated VEGFR2 is ubiquitylated, followed by epsin binding via UIM motif. ENTH domain in epsin “hooks” into the plasma membrane (PM) via PiP2. Clathrin and other endocytotic proteins such as AP2 bind to the DPW domain (Asp-Pro-Trp), while the EH-domain (Eps15 homology [EH] domain) proteins bind to the NPF domain (Asn-Pro-Phe) so that a pit is formed on the PM. Receptor-containing vesicles are endocytosed to cytosolic degradation machinery to deactivate cell signaling

Epsins were originally isolated as adaptor proteins in the clatherin-mediated endocytosis of ubiquitylated cell surface receptors.^[14,15]^ Using molecular, cellular, genetic, and mutant mouse models, we have identified that epsins modulate embryogenesis,^[16]^ angiogenesis vasculature,^[17]^ lymph angiogenesis,^[18]^ tumor angiogenesis,^[19,20]^ and cancer progression.^[21]^ Mechanistic studies have demonstrated that epsins target the Notch^[16]^ or ubiquitylated receptor VEGFR2,^[17,19,20,22]^ VEGFR3 or Wnt signaling pathway,^[21]^ and modulate angiogenesis or epithelial cell proliferation. In tumor angiogenesis, epsins bind to the ubiquitylated VEGFR2 via the ubiquitin (Ub)-interacting motif (UIM) to facilitate endocytosis and inactivate VEGFR2 signaling [Figure 1].^[19,20]^

### Epsins regulate tumor growth and tumor angiogenesis by targeting VEGFR2 signaling: role of UIM in epsins

**Figure 2:**
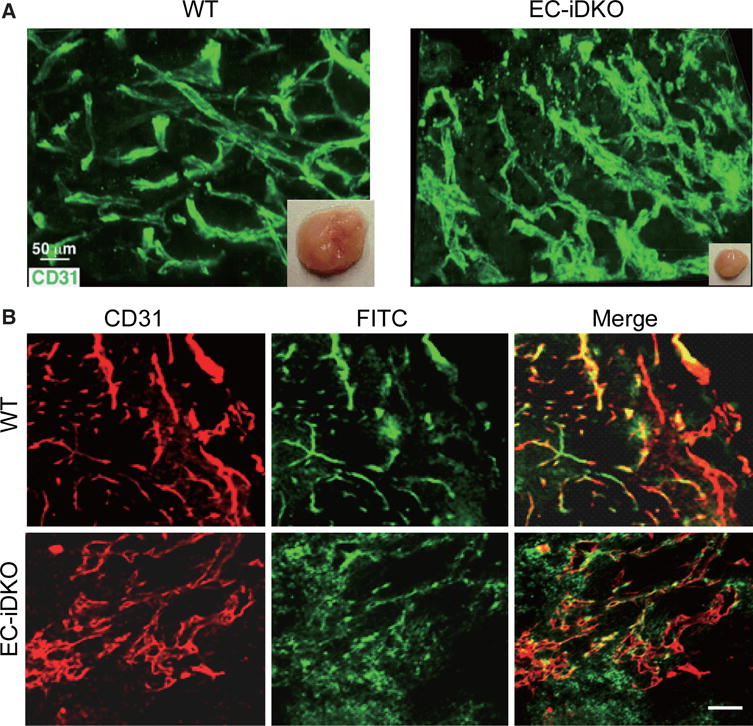
Loss of epsins in endothelial cells inhibits tumor growth by producing upregulated, but non-functional tumor angiogenesis. (A) LLC tumor is much smaller in EC-iDKO mice, accompanied by upregulated, but dilated vessels in tumors; (B) Vessel function assay: FITC-Dextran perfusion suggests that tumor vessels in EC-iDKO mice are hyper leaky. Scale bars = 50 μm for A and B. Figure 2 is adapted with permission from ref. 20. Copyright 2012 ASCI

We previously reported that the UIM-dependent binding of epsins with VEGFR2 is required for VEGFR2 internalization, degradation, and signaling attenuation in tumor angiogenesis.^[20]^ Strikingly, the UIM sequence is highly conserved in both human and mouse epsins 1 and 2, indicative of a central element in epsin function and potential clinical applications.^[20]^ In endothelial-specific loss of epsin mouse models (EC-iDKO), tumor growth is significantly inhibited in Lewis lung carcinoma (LLC), melanoma (B-16), glioma, and Tramp (Transgenic Adenocarcinoma of the Mouse Prostate) mouse models.^[20]^ The loss of epsins drastically increased not only the number of vessels but also the diameters of tumor vessels.^[20]^ Functional perfusion analysis suggests that loss of epsins leads to tumor vessel hyper leakage and dysfunction [Figure 2].^[20]^ Our data also suggest that loss of epsins modulates VEGFR2 endocytosis by upregulating its expression. The heightened VEGFR2 is anchored on the plasma membrane (PM) in EC-iDKO mice, leading to augmented VEGFR2 signaling and tumor angiogenesis.^[20]^ Because these vessels are not functional, the tumors are actually much smaller.^[20]^ A domain mapping experiment suggests that the UIM in epsins is critical for regulating the epsin-VEGFR2 interaction, and that loss of UIM in epsin 1 blocks the interaction between epsin and VEGFR2.^[20]^

### Adoption of Computer Assisted Drug Design to mimic “*in vivo*” knockout phenotype in tumor models: specificity, enrichment, and stability

To increase therapeutic efficacy, the design of the peptide is crucial. The top gear, peptide targeting specificity, peptide working mechanism *in vivo*, and peptide stability in circulation are important factors that need to be carefully considered.

#### Specific targeting

We hypothesize that if a synthetic UIM-containing peptide can be targeted to tumor vessels, it could competitively bind to the ubiquitylated VEGFR2 receptor and therefore block the epsin-VEGFR2 interaction, which could photocopy the knockout of epsins in tumor endothelial cells (TECs). Molecular modeling revealed that the UIM peptide forms a helical structure known as yeast Vps27-UIM.^[19]^ To ensure exclusive delivery of the UIM peptide to tumor vasculature, a tumor EC-homing peptide, iRGD, was conjugated to the N-terminus of the UIM peptide.^[23]^ iRGD binds to αvβ3 or αvβ5 integrin, then to neuropilin, and thus can be specifically internalized into TECs.^[24,25]^

#### Peptide working mechanism in vivo

**Figure 3:**
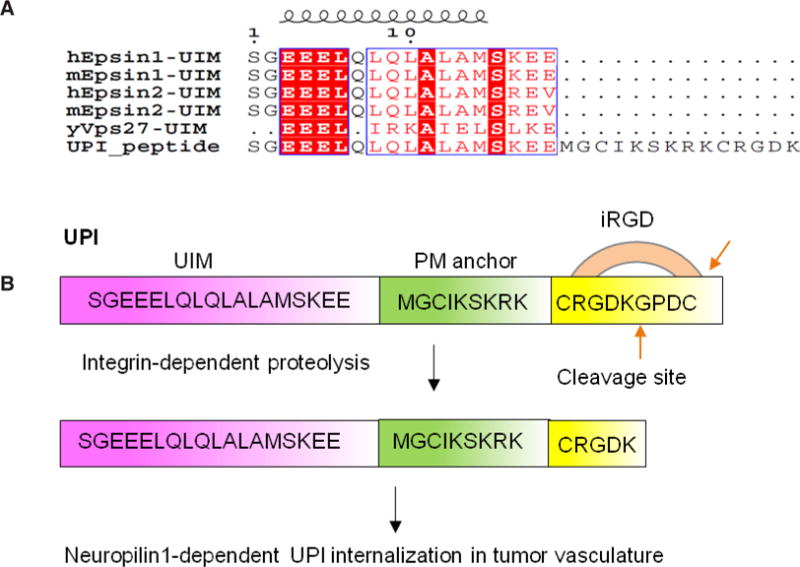
Design of the UPI peptide. (A) Alignment of human epsin UIM, mouse epsin UIM, and yeast Vps27 UIM with UPI chimeric peptide; (B) iRGD binds integrin and goes through proteolysis. The last 4 amino acids (GPDC) will be removed by circulating proteases.^[23]^ UPI peptide contains an epsin UIM, a PM targeting sequence from the Lyn kinase H4 domain, and a tumor homing sequence (iRGD). Figure 3 is adapted with permission from ref. 19. Copyright 2015 ASCI

**Figure 4:**
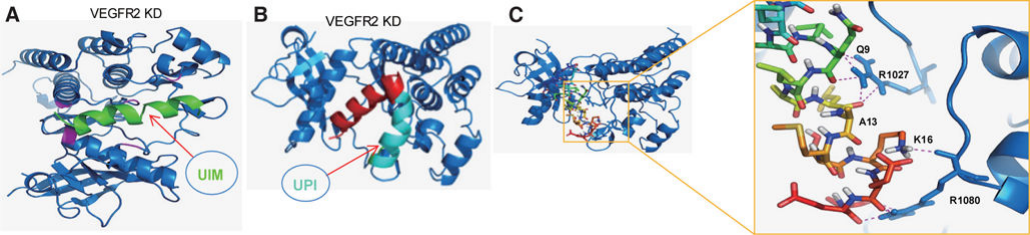
Molecular modeling to study the interaction between UIM or UPI with VEGFR2 kinase domain (VEGFR2-KD). The 3D models of UIM and UPI were predicted using the PEP-FOLD program with 200 computational simulations. The best score models of UIM and UPI were docked into VEGFR2-KD respectively using the ClusPro2.0 program. (A) Ribbon representation of the interaction between UIM and VEGFR2-KD, which are colored green and blue, respectively. The interaction residues His891, His816, Arg1022, Arg1027, and Arg1080 on the hairpin-shaped binding cleft of VEGFR2-KD are denoted in pink;[17,19] (B) Ribbon representation of the association between UPI peptide and VEGFR2-KD. In the same manner as UIM:VEGFR2-KD, UPI binds into the same binding pocket of VEGFR2-KD. VEGFR2-KD is denoted in blue. In UPI peptide, UIM is denoted in red, and the inner plasma membrane anchoring peptide and a tumor homing peptide (iRDG) are denoted in cyan; (C) Cartoon representation of the model of UIM-VEGFR2 complex. VEGFR2 is denoted in blue and shown as a ribbon; UIM is denoted in multicolor and shown as a stick (left). On the right: A close-up view of interaction residues between UIM and VEGFR2 is shown in the right panel. The key residues Q9, A13, and K16 of UIM form hydrogen bonds with R1027 and R1080 of VEGFR2.^[19]^

**Figure 5:**
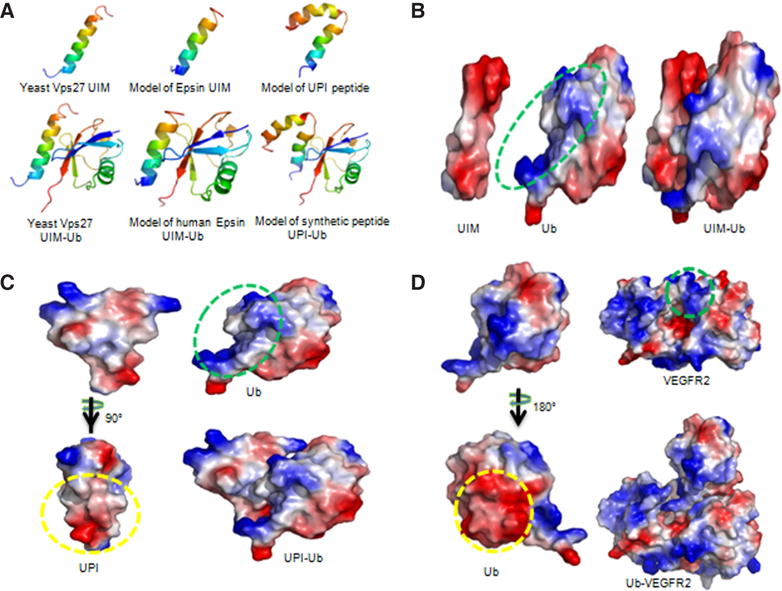
Models of UIM-Ub, UPI-Ub, and Ub-VEGFR2 complexes. (A) Ribbon diagrams show yeast Vps27 UIM interaction with Ub (left), and models of human epsin UIM-Ub complex (middle) and UPI-Ub complex (right). The NMR structure of yeast Vps27 and X-ray structure of Ub were taken from the Protein Data Bank, entries 1Q0W and 3JVZ, respectively. The top scoring models of epsin UIM and UPI (top panel) were selected and docked into Ub. The models with high scores and good topologies are shown in the bottom panel. Yeast Vps27 UIM, epsin UIM, and UIM of UPI peptide interact with Ub in a highly similar manner (bottom panel). Structures are multi-colored, with the N and C termini denoted in blue and red, respectively; (B-D) Electrostatic surface representations of UIM-Ub, UPI-Ub, and Ub-VEGFR2 complex models. Red and blue represent negative and positive potential, respectively. The proposed binding surfaces with negatively charged amino acids are indicated by yellow circles. The green circles highlight the proposed interaction surfaces of positively charged amino acids. The figures were prepared using PyMol (Schrödinger, Inc, Cambridge, MA). Note: Figure 5A is adapted with permission from ref. 19. Copyright 2015 ASCI

**Figure 6:**
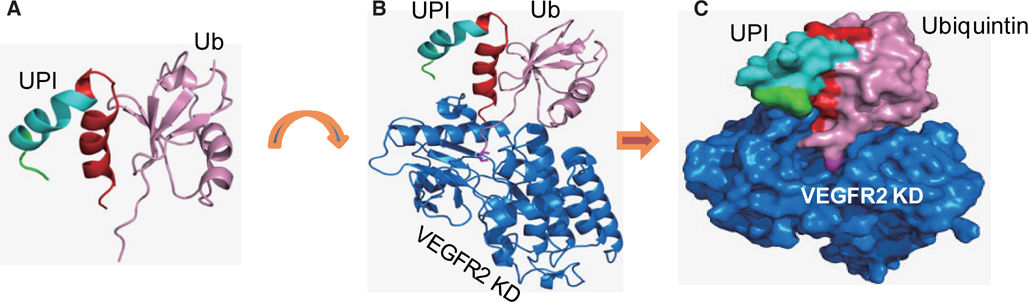
UPI-Ub-VEGFR2 KD forms a supercomplex. (A) UPI hybrid peptide (red: UIM, cyan: plasma membrane-anchoring peptide, green: tumor homing peptide) interacts with Ub (pink). (B) Supercomplex of UPI-Ub-VEGFR2. The model of UPI-Ub was docked onto VEGFR2 kinase domain (marine, PDB entry 3U6J). The UIM domain of UPI tightly binds to Ub, and the C-terminal tail of Ub (8-amino acid stretches, Gly76 side chain, magenta) inserts into the binding pocket of VEGFR2. (C) Surface representation of UPI-Ub-VEGFR2 KD supercomplex

To increase the local concentration of peptides near the PM, an inner PM-anchoring peptide from the Lyn kinase H4 domain^[26–28]^ bound to lipid rafts through palmitoylation and myristoylation sites was inserted between iRGD and UIM. The resulting peptide is referred to as UPI [Figure 3].^[19]^ To explore the specificity in the molecular interaction, we undertook docking studies and used a de novo structural prediction method to generate the atomic model for the interaction between UIM/UPI and the VEGFR2 kinase domain (KD) [Figure 4].^[19]^ Our model predicts that the unique residues Q9, A13, and K16, present only in epsin UIM but not in UIMs from a number of other endocytic proteins, play a critical role in the specific interaction with residues R1027 and R1080 in VEGFR2 [Figure 4]. Furthermore, molecular modeling revealed that interactions between the UIM helix and Ub in both UIM-Ub and UPI-Ub models are similar to the binding of yeast Vps27 UIM-Ub complex by nuclear magnetic resonance (NMR) spectroscopy [Figure 5A].^[29]^ Remarkably, the interaction surfaces of UIM-Ub or UPI-Ub and UIM-VEGFR2 or UPI-VEGFR2 are clearly complementary in terms of charges from the electrostatic point of view [Figure 5]. By binding to the Ub moiety conjugated to VEGFR2, UPI, and Ub, VEGFR2 forms a supercomplex [Figure 6]. The UPI peptide can specifically hone in to tumor vasculature and enrich itself in the inner part of the TEC’s PM as expected, which increases the therapeutic efficacy and minimizes the dosage of the peptide when used in animal administration.^[19]^

#### Optimization of peptide stability in vivo

**Figure 7:**
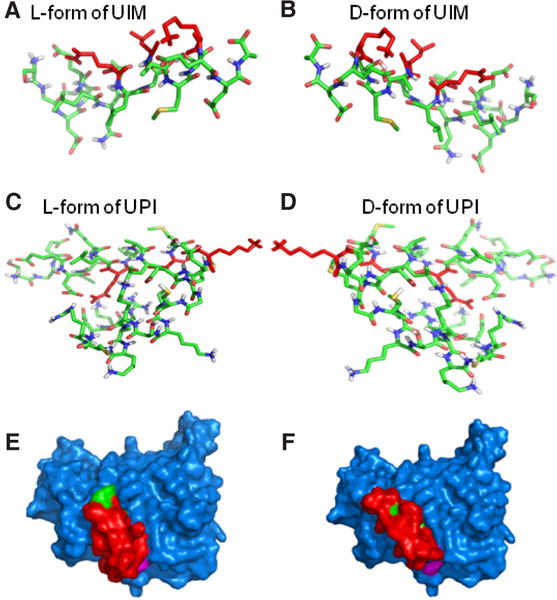
Molecular modeling to compare the interaction between D-amino acids and L-amino acids of UIM or UPI with VEGFR2 kinase domain. The 3D models of L- and D-amino acids UIM and L-and D-amino acids UPI were predicted by the PEP-FOLD program (L-form and D-form). Stick representations of the L-form of UIM (A) and D-form of UIM (B) form mirror-images of the actual structures. In the same manner, the L-form of UPI (C) and D-form of UPI (D) form mirror-images of the actual structures. (E) Surface representation of the L-form of UPI (UIM, red; anchoring peptide and iRGD, green) interacting with VEGFR2-KD (blue). (F) In the same manner as the L-form of UPI interacts with VEGFR2-KD, surface representation shows the D-form of UPI (UIM, red; anchoring peptide and iRGD, green) binds to the same binding pocket of VEGFR2 (blue)

In order to avoid the degradation of our peptide from peptidases in circulation, we used the D-isomer of amino acids to synthesize the UIM sequence, while the iRGD sequence was synthesized using the L-isomer of amino acids for efficient binding to integrin. During synthesis, the iRGD is circled by the disulfide bond of two cysteines for *in vivo* stabilization.^[30–32]^ Molecular modeling suggests that L-UIM and D-UIM show symmetric binding features to the same pocket of VEGFR2 KD [Figure 7], implying that the UIM peptide in D-isomer does not change the UIM peptide docking sites in VEGFR2 KD. Collectively, the UPI peptide is a multistep-targeting peptide to tumor vascularization and inner PM enrichment. The introduced D-isomer of amino acids in UIM and the circled iRGD can increase the UPI peptide stability *in vivo*. This design strategy could empower the function of the UPI peptide *in vivo* for better therapeutic efficacy.

### Therapeutic efficacy of UPI peptide in animal cancer models: role of UPI in tumor angiogenesis and metastasis

**Figure 8:**
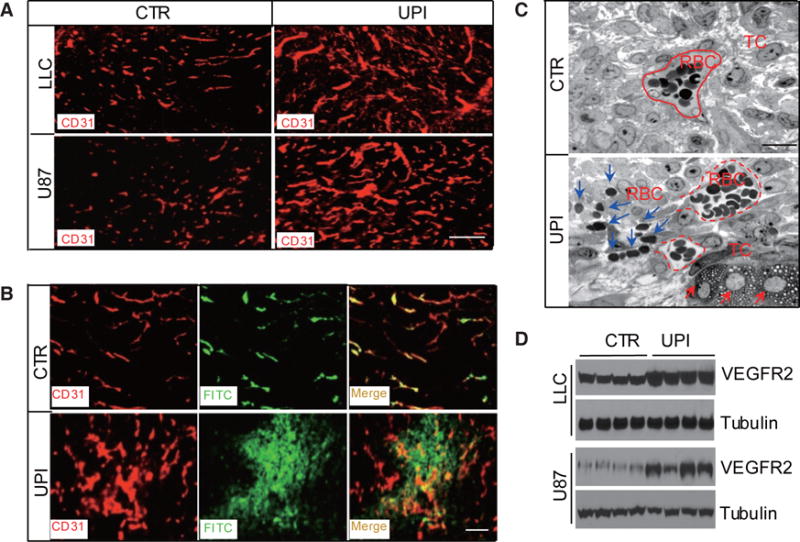
UPI peptide treatment produces upregulated but leaky vessels in tumors. (A) CD31 immunofluorescent staining for LLC or U87 tumors, showing the upregulated vessels in UPI-treated tumors. Scale bar: 100 μm; (B) Control or UPI peptide-treated s.c. U87 tumor-bearing mice were perfused with FITC-dextran for 10 min, following which the mice were killed. Tumors were fixed and processed for CD31 staining. Note that vessels in UPI-treated tumors were hyper leaky. Scale bar: 100 μm. (C) Transmission electron microscopy analysis of semi-thin sections from control and UPI peptide-treated s.c. implanted U87 tumors. Dotted red lines indicate tumor vessels; blue arrows depict red blood cell leakage from tumor vessels; and red arrows indicate dying tumor cells. Scale bar: 50 μm. (D) VEGFR2 expression in control or UPI peptide-treated s.c. LLC and U87 tumors. Figure 8 is adapted with permission from ref. 19. Copyright 2015 ASCI

UPI peptide administration can drastically inhibit tumor growth and metastasis in animal models of LLC, B16-F10, glioma brain tumor, and Tramp prostate.^[19]^ In GL261 brain tumor models, the UPI peptide can obtain a similar therapeutic efficacy and survival rate to anti-VEGF antibodies.^[19]^ More importantly, in the human U87 glioma tumor model (an immune deficient mouse model), we demonstrated that UPI peptide treatment can significantly retard tumor growth and increase the survival rate, accompanied by dysregulated VEGFR2 signaling and tumor angiogenesis.^[19]^ Mechanistically, the UPI peptide treatment generates hyper leakage vessels via upregulated VEGFR2 signaling [Figure 8] and impairs metastasis in the prostate and B-16 melanoma animal models likely due to dysfunctional tumor angiogenesis.^[19]^

### UPI peptide targeting specificity

We tested the UPI peptide specificity *in vitro* and *in vivo*. In cultured human umbilical vein endothelial cells (HUVECs), the UPI peptide treatment had no effect on other angiogenic growth factor signaling such as epidermal growth factor receptor (EGFR), platelet-derived growth factor receptor (PDGFR), fibroblast growth factor receptor (FGFR), and transforming growth factor beta (TGFb) pathways.^[19]^ We previously obtained similar results in UPI-treated mouse tumors.^[19]^ This targeting specificity may be interpreted from the length of the UIM. It has been suggested that the core components of UIMs contain 18 amino acid (aa) residues including a core Ø-XX-Ala-XXX-Ser-XX-Ac (Ø: a large hydrophobic residue; Ac: an acidic residue), which likely form a short helix embedded into different protein folds.^[33]^ Ahead of this core sequence, a short 10-aa peptide may create specificity to its binding partners. Secondly, the 3D structure of epsin-UIM may fit the binding to VEGFR2 pockets more suitably than other angiogenic receptor molecules. Third, tumor cells in general secrete more VEGF than other angiogenic substances so that VEGFR2 may have more chances to be activated and ubiquitylated. Therefore, the epsin-UPI peptide may predominantly modulate VEGFR2 signaling rather than other angiogenic receptors and signaling. This conclusion has been further confirmed by *in vitro* and *in vivo* experiments, as well as by the specific designed peptide binding assay using isolated tumor ECs.^[19]^ Interestingly, the Hrs-UIM or Eps15-UIM peptide does not show promising therapeutic efficacy in animal tumor models,^[19]^ further suggesting the specificity of the epsin-UIM peptide to VEGFR2 KD binding.

## BIOSAFETY OF UPI PEPTIDE ADMINISTRATION

We have measured the main metabolic parameters after UPI peptide administration for 3 months (10-50 mg/kg, twice a week by i.v. injection) in mice, and our results showed that UPI peptide injection had minimal side effects (data not shown). Glucose and lipid metabolism remained normal, likely owing to a relatively lower dosage used in the homing strategy. Histology and immunofluorescent staining revealed that the UPI peptide targeted to other tissues was neglected. However, whether the UPI peptide causes drug resistance in the long term warrants further investigation.

## PERSPECTIVE

**Figure 9:**
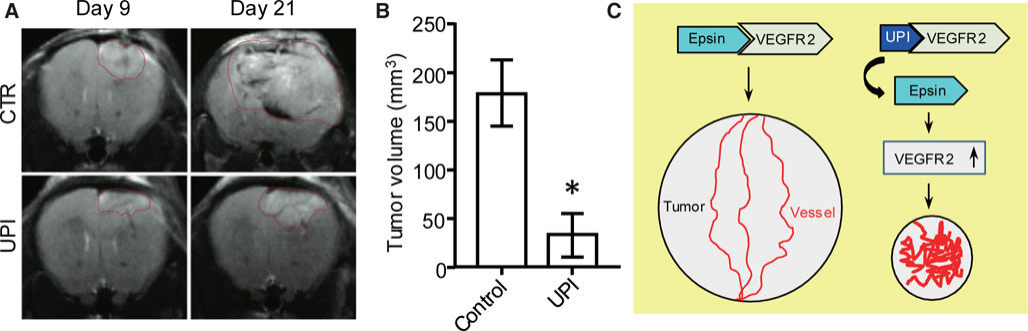
UPI peptide treatment significantly retards tumor growth in glioma tumor models. GL261 glioma cells (2 × 10^4^) were implanted to the right forebrain of C57BL/6 mice. At day 9, UPI peptide was administered by intravenous injection at 20 mg/kg dosage every alternate day. Gliomas were monitored via magnetic resonance imaging (MRI). (A) Representative MRI images. (B) Statistical analysis of tumor volume of terminal mice treated by control or UPI peptide; *n* = 5 in each group, Student *t*-test, *P < 0.001 *vs*. control. (C) Sketch of the UPI peptide therapeutic mechanism. UPI administration inhibits Epsin-VEGFR2 interaction *in vivo*, promotes non-functional tumor angiogenesis, and retards tumor growth

Serving as a potential candidate for cancer therapeutics, the UPI peptide requires more in-depth research in commonly associated cancer models such as prostate and glioma tumors [Figure 9]. Our data have shown that the UPI peptide can efficiently attenuate prostate cancer progression in Tramp mouse models and glioma mouse models [Figure 9A and B].^[19]^ Mechanistically, UPI peptide inhibits Epsin-VEGFR2 interaction *in vivo* and produces non-functional tumor angiogenesis [Figure 9C]. Specific targeting and therapeutic efficacy can be further improved by modifying the peptides, where clinical trials would then require further followup assessments. Because epsins are ubiquitously expressed proteins, it is reasonable to assume that epsins may modulate a wide array of cellular processes including cell development, differentiation, proliferation, migration, and genetics. Targeting epsins in different disease models, as well as the emergence of new technologies such as nanoparticles,^[34]^ liposomes,^[35,36]^ and CRISPR/Cas9 (clustered regularly interspaced short palindromic repeats/CRISPR associated protein 9) genome editing^[37–39]^ may also provide new directions to develop novel therapeutic agents.

## CONCLUSION

The UPI peptide is a promising compound to treat cancers. The UPI peptide can efficiently inhibit tumor growth and metastasis and specifically targets VEGFR2 signaling to create upregulated, nonfunctional tumor vessels. It is expected that the peptide may be applicable to treat cancer patients as a first or second line compound; or as an alternative replacement to the anti-VEGF antibody in patients who are resistant to anti-angiogenic therapies.
